# Raising and stabilization phase of the sit-to-stand movement better discriminate healthy elderly adults from young subjects: a pilot cross-sectional study

**DOI:** 10.1186/s40945-020-00078-8

**Published:** 2020-04-15

**Authors:** Leonardo Piano, Tommaso Geri, Marco Testa

**Affiliations:** 1 Unit of Rehabilitation and Functional Recovery, Casa di Cura “La Residenza”, via Roma 1, 12050, Rodello, CN Italy; 2 Department of Neuroscience, Rehabilitation, Ophthalmology, Genetics, Maternal and Child Health, University of Genova, Campus of Savona, via Magliotto 2, 17100 Savona, Italy

**Keywords:** Accidental fall, Aged, Geriatric assessment, Movement disorders, Posturography

## Abstract

**Background:**

The sit-to-stand (STS) test is usually included in the clinical assessment of balance and its instrumented analysis may support clinicians in objectively assessing the risk of falling. The aim of the present study was to assess if kinetic parameters of STS collected using a force platform, with particular focus on the raising and stabilization phase, could discriminate between young and older adults.

**Methods:**

Twenty-four adults (age ranging from 18 to 65 years old) and 28 elderly adults (older than 65 years old) performed STS on a force platform. Data on ground reaction forces, sway, displacement and velocity of the center of pressure were gathered during the raising and the stabilization phases.

**Results:**

elderly subjects showed significant greater global sway (146.97 vs 119.85; *p* < 0.05) and a higher velocity (vs 40.03 vs 34.35 mm/s; *p* < 0.05) of execution of STS. Between-group comparisons highlighted a greater postural sway in the raising phase (21.63 vs 13.58; *p* < 0.001) and a doubled sway during the stabilization phase (12.38 vs 4.98; *p* < 0.001).

**Conclusions:**

The analysis of STS performed on a force platform provides further information about the age-specific pattern of STS execution. The stabilization phase of STS seems to be the more challenging for functional independent older adults and should be considered during balance assessment.

Further studies are needed to confirm findings and improve generalizability of this study.

## Background

Falls in the elderly constitute one of the main burdens of health services all over the world [1, 2]. More than 30% of people older than 65 experience at least one fall per year, with an incidence that increases with age [3]. Clinical tests for the identification of people with increased risk of falling are needed to adequately develop effective fall prevention programs [4]. In addition to the classical tests currently in use [5–7] force platforms may integrate clinical assessment via the provision of quantitative data relating to parameters of center of pressure (COP) oscillations.

Posturography, recorded in different conditions (i.e. open or closed eyes), is one of the most common assessments of postural sway and balance during steady standing [8]. Even though some parameters may support clinical assessment of fall risk [8–11], the predictive value of posturography is still under debate as it lacks specificity [12, 13] and is not always suitable for detection or differentiation of healthy from unhealthy participants. However, since the majority of falls occur during dynamic tasks and transitional movements, such as postural transfers or turning around, an assessment of balance in dynamic conditions may be more appropriate [14] than static posturography.

Sit-to-stand (STS) is a functional ability significantly correlated with independence for activities of daily living [15–17] and requires coordination between the trunk and lower limbs, plus balance and stability [14]. Galli described the two main phases of STS as follows: (i) the raising phase, during which the person must move from sitting to upright posture and (ii) the stabilization phase, during which the person achieves the steady standing posture necessary for the performance of other tasks [14]. The stabilization of upright standing after the STS [18–20] requires both neurocognitive skills [21] and muscular strength [18, 19, 22]. The measurement of the STS with a force platform has been used to describe motor behavior in a specific population of elderly people [16] and some studies have demonstrated a relationship between COP parameters, individual balance, risk of falling and physical function [19, 23, 24]. However, the assessment of postural balance with posturography has not yet been investigated with specific reference to the raising and stabilization phases of the STS as critical events during which a fall may occur.

## Methods

### Aim

The aim of the present work was to assess whether the parameters used in static posturography (global sway, COP displacement and velocity) when registered during the raising and stabilization phases of STS, can discriminate between young and elderly subjects.

### Study design

The study used a cross-sectional, observational design and assessed behavior during STS and subsequent upright posture in a group of functionally independent elderly adults, with a score of at least 100 in the Functional Independence Measure (FIM) scale [25, 26], compared to a group of healthy young participants.

### Setting

The study was conducted between April 2014 and February 2016 in the Movement Analysis Laboratory at the Casa di Cura “La Residenza”, an inpatient rehabilitation center.

### Participants

Elderly adults (65 years or more) were consecutively recruited among people living in a nursing home in the vicinity of the rehabilitation center, with younger adults (aged between 18 and 65 years) being recruited among employees of the center itself.

The general inclusion criteria were absence of: neurological disease, prior orthopedic surgery of the lower limbs (such as total hip or knee replacement), or ongoing or recent (up to three months previously) disorders affecting the lower limbs or low back. As our intention was to detect early deteriorations of performance during the STS among functionally independent participants, the FIM scores had to be higher than 100 (range: 101–126) [25, 26]. Subjects were asked to sign an informed consent form to participate in the procedure. The Institutional Review Board authorized the procedure (ID: 001/2014), which forms part of a wider routine clinical assessment.

### Procedure

Before data collection commenced, anthropometric measures were gathered. Height was measured using a wall measuring tape, pelvis width, intended as the distance between anterior superior iliac spines (ASIS), was measured with a pelvimeter, and body weight with the force platform.

**Fig. 1:**
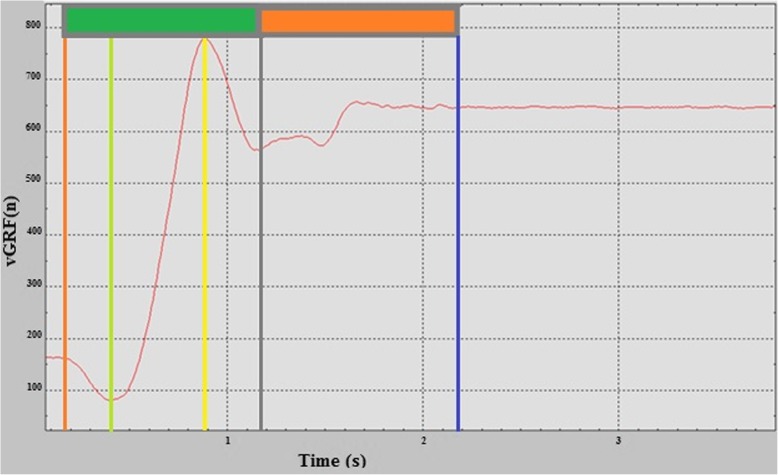
Sit-to-stand task and upright posture performed on a force platform

The distance between feet was equal to pelvis width with the knees vertically aligned to the big toes. A training session was provided before the registration of data and after 5 min of rest the participant was asked to stand up at his or her own speed while maintaining their arms crossed. The task was initiated by an auditory cue generated using an instrumental device (PocketEMG - BTS Milano) and participants stood upright looking straight in front of them for 60 s until they heard a second auditory cue (Fig. 1). Each participant performed the task three times with a one-minute rest in-between. During the task participant and examiner were alone in the laboratory to avoid distractions such as unexpected sounds.

The image refers to testing of a young subject included in the study and focuses on the key events under analysis. The x-axis shows time in seconds, while the y-axis displays the vGRF expressed in Newton.

Each vertical line corresponds to a key event as follows: orange = initiation; green = counter; yellow = Peak vGRF; black = rebound; blue = steady standing.

The small boxes on the top of the figure represent the raising phase (green box) and the stabilization phase (orange box).

### Ground reaction force

Ground Reaction Force (GRF) is defined as the force exerted by the ground on a body in contact with it [27]. It is a widely used parameter in the fields of motion analysis and biomechanics. GRF was collected using a force plate at an acquisition rate of 100 Hz.

Raw signals were digitalized with a Butterworth low pass filter with a cut-off frequency of 20 Hz.

Offset was made without any load on the platform (that is, before the participant stood on the plate). GRF data were acquired with Biomech software (BTS Milan - 2.6.107.0) and processed offline with Digivec (BTS Milan - 1.7.2.0) and SMARTanalyzer software (BTS Milan - 1.10.357.0).

The STS events were calculated according to Etnyre and Thomas [28], starting from the kinetic data corresponding to the vertical GRF, the component perpendicular to the floor corresponding to body weight in absence of external forces acting on the body (for instance during gait or running).

We identified the following events:

- *Initiation*: the first deflection of vertical GRF following the start of the acquisition time
- *Counter*: the lowest value in the vertical GRF
- *Peak vGRF:* the greatest value of vertical GRF, following the seat off, belonging to the ascending phase, when the participant reaches an upright posture [28]
- *Rebound:* the lowest value of the vGRF following the peak vGRF.

*Steady standing (SS):* the first event following the rebound when the recording levels to normal postural sway. This event was identified through visual assessment since it was found more reliable than any other algorithm [28]. The STS movement was divided into two stages as proposed by Galli [14].

Events were analyzed using SMARTanalyzer software (version 1.10.357.0 – BTS Milan). We started from the peak vGRF and counter, which indicate respectively the maximum and the minimum value of vGRF during the whole acquisition time.

Accordingly, the rebound event was identified via the minimum of the curve following the peak vGRF, while the counter event was identified with the maximum.

The following phases represented the major interests of our analysis.

- *Raising phase:* the transitional period during which the body moves from a sitting to an upright position [29]. As part of our aim to distinguish elderly from younger participants, the procedure divided the raising phase into two parts: the first one lasting from initiation until peak vGRF (T1), and the second one from peak vGRF until rebound (T2). Sipko et al. proposed a similar approach using the vGRF to identify the subsequent events in their study about patients with chronic low back pain [30].
- *Stabilization phase:* the period between rebound (upright posture) and the achievement of the SS.

To extend accuracy and provide quantitative data to support visual assessment, we analyzed a later phase, between the ninth and tenth second after the start of SS. Subjects were considered to be in a steady posture if the variability of the vGRF (expressed in Newton) did not exceed a single standard deviation from the mean of the three repetitions.

### Platform data - COP

COP is the point of application of the GRF vector [27]. COP displacement and velocity [31, 32] were recorded with a force platform (Kistler CH -9286A). Biomech (BTS Milan - 2.6.107.0) and Digivec softwares (BTS Milan - 1.7.2.0) were used to calculate the displacement of COP.

Using Sway software (BTS Milan), that provided real-time information during the whole acquisition time, we derived COP parameters relating to the 60 s of the entire trial.

With SMARTanalyzer software, we calculated the length of COP sway during the raising and stabilization phases by interpolating the GRF with the events defining each specific stage (for example, peak and rebound to define T2).

The COP displacement (global sway) is the common length of the trajectory of the COP sway calculated as a sum of the point-to-point distances. A normalization to body height was computed to control for the influence of the variable height on the global sway.

COP displacement was also collected during raising and stabilization phase. Velocity was calculated as the ratio between the COP displacement and computation time.

Mean and standard deviation of the repetitions were calculated for both COP displacement and velocity.

### Statistical analysis

Statistical analysis was performed using R software [33].

Normal distribution was tested through the Shapiro-Wilk test and q-q plots. The two groups were then compared using the Wilcoxon signed-rank test. The level of significance was set at 5% (*p* < 0.05).

## Results

Twenty-four adults (18 F, age: 33.1 ± 10.2 years, range 18–60) and 28 elderly adults (21 F, age: 75.3 ± 7.3 years, range 65–90) were included in the study. All participants completed the procedure for a total of 156 movements acquired and analyzed. A summary of the main sample’s characteristics is presented in Table 1.

**Table 1:** Sample Characteristics

	YOUNGER ADULTS (mean +/− SD)	ELDERLY (mean +/− SD)
Gender, M/F	24 (6 M/18 F)	28 (7 M/21 F)
Age, y	33.13 (10.53)	75.29 (7.29)
Height, cm	168.36 (10.62)	161.42 (10.22)
Weight, kg	67.08 (10.75)	71.54 (18.22)
Pelvis width, mm	195 (15)	203 (19)
FIM	125.79 (1.02)	112.32 (5.86)

Anthropometric and personal data are reported as means and standard deviations

### COP spatial-temporal parameters

Global sway was found to be significantly greater among the elderly: normalized data displayed 146.97 for elderly compared to 119.85 for the young subjects (*p* < 0.05).

Mean COP velocity was higher in the elderly group (40.03 ± 8.27 mm/s) compared to young subjects (34.35 ± 6.85) with a statistical significance (*p* < 0.05).

Raising and stabilization phases displayed greater differences compared to the acquisition time as a whole. In fact, the stabilization phase seemed to display most relevance since postural sway was almost 2.5 times greater among the elderly in comparison to younger people (12.38 vs 4.98; *p* < 0.001 – Table 2).

**Table 2:** Between Groups Comparison of the COP Displacement during Global Sway, Raising and Stabilization phase

Variable	YOUNGER ADULTS (mean +/− SD)	ELDERLY (mean +/− SD)	*P* value
Global sway	119.85 (27.81)	146.97 (39.52)	*P* < 0.05
Raising phase sway	13.58 (2.88)	21.63 (10.71)	*P* < 0.001
Stabilization phase sway	4.98 (16.39)	12.38 (6.45)	*P* < 0.001

Between groups comparison regarding COP displacement during the global task, and the raising and the stabilization phase. Data were normalized and expressed as % of body height

### Time to peak vGRF

The elderly group reached the peak vertical GRF significantly later compared to the group of young participants (Table 3).

**Table 3:** Key Events of the STS

Variable	YOUNGER ADULTS (mean +/− SD)	ELDERLY (mean +/− SD)	*P* value
Time to Peak vGRF, ms	1073 (216)	1312 (392)	*P* < 0.05
Peak vGRF, normalized (% BW)	122.63 (8.18)	116.14 (6.2)	*P* < 0.001
Rebound – upright standing, ms	1573 (295)	1816 (398)	*P* < 0.05
Steady standing – SS, ms	2814 (759)	2116 (381)	*P* < 0.001
SD 10 s after SS	0.88 (0.32)	1.19 (0.50)	*P* < 0.001

A summary of the key events of the task. Level of significance 5%;

% BW = percentage of body weight; Peak vGRF (N) was normalized to body weight

However, elderly subjects gained SS earlier than younger ones, although in the elderly group there was greater variability while reaching subsequent phases of upright posture: comparison between the performance of the two groups at ninth to tenth second after attainment of SS showed excess of one standard deviation with significant difference (*p* < 0.001). Data refer to the mean of three trials.

## Discussion

To our knowledge, our study is the first to focus on specific stages of the sit-to-stand movement providing further information that may identify an early deterioration of the motor performance [34–36].

The main finding was that segmentation of the STS into the sub-phases proposed by Galli [14] highlighted significant differences between groups regarding COP displacement, with elderly subjects displaying greater postural sway during both raising and stabilization phases, thereby suggesting a more unstable performance in reaching and maintaining SS.

Similarly, results from the global acquisition data pointed out a less effective performance of the elderly group who displayed greater displacement and higher velocity of their COP during the whole acquisition time.

The longer global sway of the elderly supports the hypothesis that elderly are less effective in the execution of the STS [37]. The higher mean velocity of COP observed in this study is consistent with previous studies which defined this parameter as one of the most important to differentiate healthy young from elderly subjects, relating the postural instability of elderly to an increased risk of falling [10, 38, 39]. Taken together, all these parameters could be useful to assess the efficiency of postural stability of a person facing the specific task of STS.

In addition to these significant differences between the groups, the more than two-fold increase in postural sway during the stabilization phase among the elderly may support clinical relevance of the stabilization phase as the moment in which the kinetics of STS movement are most likely to be altered.

Interestingly, it was observed that elderly participants achieved SS earlier than younger ones, in contrast with data reported by other authors: this finding may be interpreted as a need for the elderly to gain upright posture more quickly in order to save on muscle involvement [14, 18, 40]. On the other hand, it is also possible that visual assessment of the initiation of SS may have introduced a bias: future studies need to adequately synchronize testers to eliminate any such fault. In contrast, the longer time needed for the elderly to achieve peak vertical GRF may indicate either a slower reactive strategy or global muscle weakness: these issues are coherent with previous studies which highlighted how impaired muscles and muscle fatigue can influence performance in the elderly [41, 42]. Further, this fact seems to confirm the importance of advising elderly people to perform muscle strength training of the lower limbs, especially the gluteus maximus, quadriceps and other antigravity muscles, in order to best maintain functional independence. The clinical implementation of these results seems to indicate the reduction of the speed of execution of lower limb strengthening exercises, such as squats, during specific fitness programs for the maintenance of functional independence in the elderly [43].

Two possible confounding factors have also to be taken into account: firstly, the starting position was left unchecked, aside from limb shaft alignment. Secondly, it is possible that differences between our two groups were confounded owing to excessive similarities in terms of functional independence with the elderly group containing subjects exhibiting structural and functional changes too subtle to alter their motor performance. Nevertheless, the higher mean velocity of COP observed in this study has been linked with postural instability of elderly [8] that, in turn, leads to increased risk of falling [10]. Moreover, the longer global sway in the elderly group supports the hypothesis that the elderly are less efficient during performance of STS [37]. Taken together, all these parameters could prove useful in the assessment of efficiency of postural stability in an elderly person facing the specific task of STS.

The present experiment has some methodological issues that require attention: for example, the use of the FIM scale as single indicator of the patient’s functional status. Different results might also have resulted if data on other potentially confounding factors, such as sarcopenia, frailty status, comorbidity and drug intake had been included in the study.

In addition, our sample size of elderly subjects was small, with all subjects recruited from the same nursing home, facts that might have hindered the extrapolation of our results to wider groups of elderly adults, including those who live alone. We suggest that future studies in this area should consider the present result as a reference value for power analysis and should concentrate on collecting data on potential confounding factors, such as concomitant health conditions.

An additional methodological concern is that of the biomechanical conditions necessary to perform STS: previous studies regarding STS placed subjects in a starting position of 90 degrees flexion of hip, knee and ankle joints [26, 36], but this does not correspond to a physiological sitting position and proves too challenging to many subjects with physical impairments such as severe muscle weakness and/or a limited range of motion. Since our aim was to transfer our study findings into clinical practice we decided not to oblige participants to perform an excessively standardized movement [20]: people could choose their best preferred motor strategy and their preferred velocity. Despite this possibly constituting a bias and subsequent limitation of the study [19], we thought it would best represent the inherent variability of the movement [44]. The only indication we imparted to subjects was to maintain their arms crossed and to adopt a starting position with patellae and big toes vertically aligned. This position allowed the necessary advancement of the tibia for efficient STS to be initiated and is closer to the mechanical conditions present as an individual rises from their chair under normal circumstances.

A further technical issue regards the duration of the task: our findings seem to suggest that a global acquisition time of 10 s of posturographic registration of STS is enough to reveal differences, thereby reducing the impact of fatigue and discomfort provoked by longer upright posture.

Recruitment bias was contained by the use of inclusion and exclusion criteria. The bias related to the experimental procedure was reduced via accurate set-up of laboratory equipment and standardized positioning of the participant.

The small sample size does not allow for generalization of our results and their translation into clinical concepts should be made cautiously. However, its clinical relevance and the possible application of this pilot study should be assessed in relation to the continuous development of easily accessible and portable technologies, such as smartphones and tablets, which could facilitate clinicians’ use of these findings in a variety of clinical rehabilitation environments.

The analysis of STS using force platforms could be used to support clinical evaluation of balance, providing additional information regarding: (i) the main strategies adopted by subjects during performance of STS, and (ii) the degree of postural impairment as measured using COP displacement during two specific moments of the functional movement as a whole.

Since an impaired raising phase seems to be associated prevalently with loss of muscle strength [45], whereas the stabilization phase may be more altered in people with restricted ability to manage the postural changes, clinicians could use this assessment to calculate the role of muscle weakness in the execution of STS and in balance maintenance.

The procedure described in the present manuscript may represent an innovative way to assess balance providing sensitive information unavailable from other clinical tests that could prove useful in the detection of balance impairments in functionally independent subjects.

From a clinical perspective, the results of our study may in time prove to be clinically relevant even though caution is required here since at the present time no firm evidence exists regarding the correct management of platform data and any clinical applications therefore remain to be defined.

Further work will be necessary to evaluate the validity and reliability of our study, including some of its methodological features, with the aim of providing more finely detailed information on motor performance during STS. Our study attempted to decrease bias via the use of stable, objective methods, such as trial repetition and auditory cues.

If further studies confirm our findings, clinicians will have a new instrument at their disposition for the assessment of balance while the ever-expanding availability of new technologies may facilitate the extrapolation of our results and support the application of the test to different study populations.

## Conclusions

The results of the present study provide some additional knowledge of the potential use of a functional test such as STS in the assessment of balance. The transitional period of the task (that is, the raising and stabilization phases) might be relevant in discriminating the elderly from younger subjects, with a higher displacement of the COP among the elderly, which may in turn be related to poorer competence in managing an upright posture. Above all, the stabilization phase seems to represent the moment of specific relevance during STS necessary for the distinction between elderly and young subjects. Although our results indicate the possibility of integration of STS into the clinical assessment of individual balance ability, further studies correlating STS outcomes with validated balance assessment tools in wider samples of participants are required to improve both the internal and the external validity, thereby permitting extrapolation of our results to clinical practice.

## Data Availability

The datasets used and/or analysed during the current study are available from the corresponding author on reasonable request.
